# Unifying Outpatient Practices to Redress Structural Racism in an Urban Health System

**DOI:** 10.1001/jamahealthforum.2024.5520

**Published:** 2025-02-21

**Authors:** Sofia R. Schlozman, Margaret Smirnoff, Ann Scheck McAlearney, Carol R. Horowitz, Lynne D. Richardson, Radhi Yagnik, Nina A. Bickell

**Affiliations:** 1 UCSF School of Medicine, University of California, San Francisco; 2 Department of Environmental Medicine and Climate Science, Icahn School of Medicine at Mount Sinai, New York, New York; 3 Department of Family and Community Medicine, The Ohio State University, Columbus; 4 Institute for Health Equity Research, Department of Population Health Science and Policy, Icahn School of Medicine at Mount Sinai, New York, New York; 5 Institute for Health Equity Research, Department of Emergency Medicine, Icahn School of Medicine at Mount Sinai, New York, New York

## Abstract

**Importance:**

There is a strong and increasing focus on redressing structural racism in health care systems. Structural racism persists by separating clinical care sites that treat patients of racial and ethnic minority groups who are disproportionately covered by Medicaid from sites that treat patients who are White and disproportionately covered by commercial insurance. Practice unification refers to efforts to eliminate this form of segregation.

**Objective:**

To define and investigate the facilitators, barriers, and the effects associated with unification of outpatient practices to reduce structural racism in a large urban health system.

**Design, Setting, and Participants:**

This qualitative study used semistructured interviews conducted within a large urban health system in New York from February to October 2023. Trained researchers interviewed clinical and administrative leaders of outpatient clinical practices that were pursuing unification, and health system leaders overseeing multiple practices.

**Main Outcomes and Measures:**

Thematic analysis was used to identify facilitators of and barriers to unification, challenges and benefits after unification, and persistent dimensions of segregation within clinics that had nominally unified. These insights were used to create a framework for the unification process.

**Results:**

The thematic analysis included qualitative information from 5 administrative leaders, 12 clinical leaders, and 6 health system leaders, and found that unification facilitators were financial benefit, relocation to new facility spaces, and advocacy by leaders and trainees, while barriers were financial concerns, space constraints, and physician and staff attitudes. After attaining and experiencing some degree of practice unification, interviewees reported financial gain, more support staff, perceptions of greater equity, better educational experiences, and increased practitioner and trainee satisfaction. Challenges reported after unification were changes in staff roles, financial concerns, patient dissatisfaction, and difficulties interfacing with segregated practices within the health system. Partially unified practices maintained dimensions of segregation, by practitioner, payer, and/or scheduling/time (temporal segregation).

**Conclusions and Relevance:**

This qualitative study found that outpatient practice unification was perceived to be a financially and equity-driven process with multiple dimensions. However, not all of the unification procedures had been completely implemented. These findings indicate that successful unification of outpatient practices in a large urban health care system requires attention to multiple dimensions, as well as overcoming challenges regarding finances, facility space, reimbursement policies, and patient and staff satisfaction.

## Key Points

**Question:**

What are the facilitators, barriers, and effects associated with reducing health care segregation by unifying outpatient practices within a large urban health system?

**Findings:**

This qualitative study analyzed information from 23 interview participants and found that clinical, administrative, and health system leaders characterized practice unification as a financially motivated and equity-driven process with several financial, space, staffing, advocacy, and facility benefits and drawbacks.

**Meaning:**

Successful unification efforts require attention to factors such as health system finances, facility space, reimbursement policies, patient and staff attitudes, and advocacy.

## Teaser

This qualitative study investigates the benefits and challenges of addressing health care segregation by unifying outpatient practices within a large urban health system.

## Introduction

There is a strong and increasing focus on redressing structural racism within health care systems.^1,2,3,4^ Despite the abolition of legal segregation decades ago,^5^ structural racism remains evident in de facto health care segregation.^6^ Segregation often results from redlining practices affecting neighborhood racial, ethnic, and socioeconomic composition, and health and quality of care.^7,8,9^ It is further exacerbated when patients covered by Medicaid are treated at different health care sites and/or by practitioners than patients covered by commercial insurance.^6,10^ Because patients of racial and ethnic minority groups are overrepresented in the Medicaid system, segregation by insurance can lead to de facto health care segregation.^11,12^ Moreover, because Medicaid reimbursement rates are usually lower than commercial rates, these policies may reduce health care access and quality for patients of minority groups within a health system.^13,14^

Practice unification refers to efforts to eliminate segregation by race and ethnicity through combining multiple care sites into single care sites that do not differentiate between patients by insurance type. As part of a broader antiracism action plan, an urban health system in New York, New York, is undergoing institution-wide efforts to unify care sites. This study sought to investigate the barriers, facilitators, and positive and negative effects associated with unification efforts between ambulatory practices that unified successfully and those that were only partially unified.

## Methods

This study was reviewed and approved by the institutional review board of the Icahn School of Medicine at Mount Sinai. Written informed consent was obtained from each participant prior to the interview; participants agreed to be recorded and to have their verbal responses qualitatively analyzed after deidentification. We followed the Consolidated Criteria for Reporting Qualitative Research (COREQ) reporting guideline.

### Study Participants

We conducted semistructured interviews with clinical, administrative, and health system leaders at multiple unified ambulatory practices across a large urban health system. The health system comprised 8 hospital campuses, including more than 7200 physicians throughout the 5 boroughs of New York City, Westchester, and Long Island. Ambulatory leadership identified all 13 unified practices and reached out to engage these practices’ clinical and administrative leaders to participate. Interviewees were chosen based on their position. Each participant self-described their demographic characteristics, including race and ethnicity; age was not considered.

### Data Collection

Trained researchers (N.A.B. and M.S.) conducted 30- to 45-minute interviews using internet videoconferencing software between February and October 2023. Questions focused on care delivery and financing, the current status of unification of the practice, and the barriers, facilitators, challenges, and benefits associated with unification. Participants’ demographic characteristics, including race and ethnicity, were self-described and reported. Interviews were recorded, transcribed, and deidentified.

### Data Analysis

We performed qualitative thematic analysis with a modified grounded theory approach that included open and focused coding.^15,16^ A preliminary codebook was developed based on interview guide questions and discussions with the research team. Interview data were analyzed using Dedoose, version 9.0.90 (SocioCultural Research Consultants LLC), a cloud-based software application designed for rigorous analysis of qualitative and mixed-methods data. A single researcher (S.R.S.) then coded all transcripts while meeting regularly with the team to discuss emerging themes, address questions and challenges about the codes and themes, and receive feedback about the application of codes. The codebook was iteratively modified following each discussion, and transcripts were recoded to incorporate new codes as they were developed.

We used matrices and data displays to present emerging ideas and begin shaping a framework for the unification process. Using this iterative analytic process, the team established key features of unification and developed definitions for persistent dimensions of segregation described by the participants. We defined practices that did not accept patients covered by Medicaid as completely segregated; practices segregated by practitioner, payer, or scheduling/time—but not by care site—as partially segregated; and practices where all insured patients are seen at the same care site and by the same practitioners during the same days and/or time periods as completely unified. Using these definitions and findings, we assembled a framework representing a high-level view of the unification process as described by study participants.

## Results

The analysis comprised interview data from 23 participants (12 [52%] females and 11 [48%] males), including 5 administrative leaders, 12 clinical leaders, and 6 health system leaders whose positions spanned multiple departments and/or practices. This study sample represented 8 specialties and all 13 unified practices within the health system. Table 1 lists additional participant characteristics. Table 2 indicates the status of unification, as reported by participants, for each practice.

**Table 1.** Demographic Characteristics of the Interview Participants

Characteristic	Participants, No. (%)
Participants, No.	23
Female	12 (52)
Male	11 (48)
Birth country	
US	15 (65)
Other country	8 (35)
Race and ethnicity^a^	
Asian	5 (22)
Black/African American	2 (9)
Hispanic/Latino	3 (13)
White	10 (43)
Multiracial	2 (9)
Other	1 (4)
Education	
Graduated fellowship training	8 (35)
Graduate degree	14 (61)
College degree	1 (4)
Time since education completed , y	
0-9	4 (17)
10-19	8 (35)
20-29	4 (17)
≥30	7 (30)
Specialty	
Cardiology	4 (17)
Gastroenterology	1 (4)
General medicine	4 (17)
Oncology	3 (13)
Ophthalmology	1 (4)
Pulmonary	2 (9)
Rheumatology	3 (13)
Transplant	3 (13)
Cross-departmental role	2 (9)

^a^ Self-described; other race or ethnicity was completed by the participant in a free-form text field.

**Table 2.** Status of Unification Process Across Clinical Outpatient Practices

Unification status	Practices, No.	Definition
Completely segregated	2	Does not accept patients covered by Medicaid.
Partially segregated		
By practitioner/payer	4	Patients covered by Medicaid are more frequently seen by trainees, not attending physicians, or are seen by a subset of practitioners who accept Medicaid.
By scheduling/time	4	Patients covered by Medicaid are intentionally scheduled for different days and/or times than other patients.
Completely unified	7	All insured patients are seen in the same location, by the same practitioners, and on the same days and/or in the same time periods.

Facilitators of and barriers to unification refer to factors that made unification efforts easier or more difficult, respectively. Benefits and challenges of unification refer to positive and negative impacts that occurred after some degree of practice unification occurred. Table 3 presents primary facilitators, barriers, benefits, and challenges, alongside representative participant quotations.

**Table 3.** Domains and Sample Quotations

Domain	Representative quotation
**Facilitators of unification**	
Financial incentive	We’ve been a merged practice since 2011…our ambulatory cancer center became what’s called an article 28 facility, so a hospital-based practice as opposed to an FPA. Prior to that we had an FPA practice, and then we had a fellows’ clinic, but in 2011, that changed. And you know the guiding principle for that [merger] at the time was mostly around sort of payment structures and models as it related to cost of drugs. I think it was mostly around pharmacy benefit, because of, you know, the chemotherapy drugs that we give are expensive. And there was more of a discounted rate if we were able to provide that on the hospital side as compared to the FPA side.
Change in space or practice structure	…I have to say it [unification] happened at a very, I don’t want to say a good time, because we were coming out of the pandemic, but because we had been closed, it allowed the restructuring. It was a little easier to work it in because we were doing new templates. We were basically starting from scratch.
Advocacy by leaders and trainees	Our division chief, you know, he really had really excellent foresight when it came to that [clinic unification], because, you know, he had been talking about it even before the pandemic.
**Barriers to unification**	
Financial concerns	Could it [the practice] potentially be even more integrated? Yes, it could. We’d have to… talk about the financial implications. While the global collections are higher [in a unified practice], we have to understand that the professional collections are lower. So then, let’s think of, “What’s attractive to the physician recruit?”
Lack of space	It [the possibility of unification] is in the distance only because that’s where the space is going to be developed. You know, it [unification] is definitely talked about between medicine leadership and the institutional leadership, it’s just…you know, the whole place is landlocked in terms of clinical space.
Physician and staff attitudes	I think provider buy-in… it’s actually one of the barriers. And what I mean by that is that I think initially in onboarding some of them [the providers], the mentality of “I’m in the faculty practice” vs “I’m coming to the clinics.” What are the expectations? [Some doctors are] thinking that the patients are 2 different types of populations… and they maintain that. And don’t see…how those groups can be integrated. And it’s just a very traditional, you know, way of how medicine used to work.
**Benefits after unification**	
Greater staffing support	The staff we had at [previously more segregated clinics], we were able to bring them to [newly unified clinic]. So, we were suddenly well staffed. We had 2 LPNs, 2 MAs, and they gave us 2 RNs on top of it, which we didn’t have before. Plus, we kept our 2 medical secretaries. And we got a clinic administrator which was great…So suddenly, there’s all this support staff, plus prior authorization. And now people are being roomed in 2 minutes or 5 minutes, if it’s a new patient or whatever, and on time. And it’s just bang, bang, bam, you know, just wow!
Perceptions of greater equity	I think it’s wonderful that there’s no distinction made between patients that get cared for. Period. I mean, there are individual differences with clinicians. We all, you know, there’s the art of medicine as well as the science. We all practice the art a little bit differently. But if you’re a patient of Dr X, you’re going get treated in the way Dr X generally treats you, whether you have Medicaid or commercial insurance. So, I think that’s a very important step in advancing equity in our division. And I hope it happens in others.
Increased practitioner and trainee satisfaction	The other thing that I will say is that every time we do this [unification], people are nervous about it, but afterwards, almost without exception, people are like ‘This is the best thing we ever did. It’s so much easier. You know, the staff understands what our needs are, and any patient I see in the hospital can just come see me afterwards. I mean the amount of positive feedback we get from the staff and faculty.
Financial gain	For the institution, even though the ratios of the staffing are a little higher, it [unification] is also an improvement because you have the split bill. The adding of the professional vs the technical. It’s like twice what you were getting as a global deal. Financially, until the insurance changes the rules, it’s a beneficial piece.
Better educational experience	[Segregated clinics] led to a bad educational experience because that clinic was overburdened with the high volume of patients. Unification of the clinics...happened a number of years ago, and the main impetus back then was really 2-fold, number 1, to create a single tier system, to treat all kinds of patients in the same structure which included a subspecialized type approach to oncology, which is, as you know, state of the art… And so we tried to move away from having one format for [faculty practice] and one format for the clinic. And the other, which was just as important was to create a teaching experience for the fellows which was closer to what our top-tier competitors were offering for education...where the fellows really learn hematology, oncology on subspecialized and participate in the care of patients at subspecialized programs.
**Challenges after unification**	
Changes in staff roles	All the staff move from kind of school-based FPA staff to hospital staff, which are union with very strict roles, scope of work, etc. And that has been challenging for these practices. They’re used to having, you know, a sort of righthand person that can do everything. And in our practices, there are limitations on that. And so, we have a whole work plan around making sure that that all gets straightened out. And I think that takes the doctors time to adjust to this.
Financial concerns	So, the professional revenue goes down in an Article 28, and so the piece of the revenue that goes to the division, the department, the school of medicine, goes down because you’re now billing on the technical component. So that piece affects the school, affects the division. It’s not a huge amount of money, but it does affect the school and the division and the department negatively.
Patient concerns	What’s very interesting is that I had a few patients who were aghast at the idea of being seen in a clinic setting. I had several patients say to me, “You mean I have to sit with those people.” And I looked at them, and I said, “Those people are also my patients.” And a few of them did not see me for a little while, but they have come back to sit with those people and be seen. But isn’t that something? It was just a horrible thing.
Interfacing with segregated practices	Well, the challenge is that because we see everyone, it is difficult when we need to send our patients to see specialty-level clinicians, in getting patients in to see other folks in a timely manner… I often truthfully don’t know what insurance someone has until it becomes a problem. And so, when I then go to refer a patient out to see someone, I’m like, “send them to this person” because it’s just the person I send to, and then it’s like, “Oh, no, I can’t. They have Medicaid, right?”

Abbreviations: FPA, faculty practice associate (commercially insured practice); LPN, licensed practical nurse; MA, medical assistant; RN, registered nurse.

### Facilitators of Unification

Three factors helped drive clinic unification: financial incentives, changes in space or practice structure, and advocacy (by leaders, trainees, or medical students). Because Medicaid reimbursements are consistently and substantially lower than those of commercial insurance,^17^ efforts to ensure financial stability were critical components of unification discussions. Specialties with substantial reimbursements from procedures and from Medicare described these as financial enablers of their practices’ unification.

Financial incentives related to the 340B Program, a federal drug-pricing program that allows qualifying hospitals and clinics to purchase expensive outpatient drugs at reduced prices, were key to unification. One participant stated that 340B-related enhancements are the “whole reason that fueled… integration [unification]” and that fully unified specialties all rely on expensive pharmaceuticals: “Oncology has chemo drugs, Hepatology has liver drugs, Rheumatology has their specialty meds.”

Other participants framed the financial gain associated with being a New York State Article 28−licensed institution as a facilitator of unification. Article 28, a New York State−specific policy, provides additional reimbursement for health care services performed in accredited facilities, which enables higher rates of reimbursement for hospitals given that both the facility and physician fees are paid per visit. Because Article 28 guidelines also require that clinical spaces meet certain structural standards to receive accreditation, participants cited the importance of an available Article 28−compliant space in facilitating unification. One participant noted that when the unification occurred, “there was already planned construction for that space,” which enabled the unified practice to “accommodate the Article 28 needs,” and receive additional reimbursement without a “heavy lift.”

Participants also discussed the impact of other types of restructuring on the feasibility of unification. One practice, which was previously completely segregated, reopened with a more unified structure following the COVID-19 pandemic. In this case, the administrative leader cited the ability to “start from scratch,” as a key facilitator in adjusting to new policies and structures associated with unification.

Also, participants suggested that unification efforts are heavily driven by specific advocates within the practice undergoing unification. Some advocates were department or hospital leaders who recognized unification’s importance from an equity standpoint and promoted top-down changes. Others included medical students, residents, and fellows who viewed opportunities to improve care through integration and “created enough noise” to accelerate unification.

### Barriers to Unification

Financial concerns also serve as a barrier to unification. Participants argued that although overall reimbursement may be higher in unified practices because Article 28 requires billing both facility and practitioner fees, physician payments are reduced. Participants blamed these reductions for lower physician salaries and less-attractive packages for physician recruits.

Interviewees also cited lack of space as a major barrier to unification. Many expressed that, while they acknowledged the importance of unification, their practice did not have access to a clinical space large enough to accommodate all the patients that needed to be served, particularly to meet Article 28 compliance requiring larger space (eg, examination rooms ≥100 ft^2^). These issues were heightened by the health system’s urban location, which is “landlocked in terms of clinical space,” making expansion particularly difficult.

Some participants also noted that some individual physician’s and staff member’s attitudes discouraged unification efforts. Some interviewees felt that some physicians preferred a segregated care model, viewing it as the “traditional” way medicine should function. In other cases, participants felt that physicians at faculty practices preferred not to adjust to new policies, or assumed that existing patients would be dissatisfied with structural changes related to unification.

### Benefits of Unification

Major benefits to unification cited by participants included financial gain, greater staffing support, improved practitioner and trainee satisfaction, perceptions of greater equity, and better educational experiences. In several practices, the financial benefits associated with implementing Article 28 and the 340B funds used to hire additional staff members allowed for greater support. In other cases, staff members from faculty practices, which tended to have more administrative support, were brought on after unification, enabling hospital-based clinics to benefit from more experienced or a larger number of support staff. The increase in staffing alleviated pressure on practitioners and trainees to manage administrative tasks, leading to an improved and more fulfilling educational experience and, more broadly, improved efficiency within newly unified practices.

Several participants noted the logistical benefits that followed unification, which made providing care “much easier.” Physicians no longer had to consider a patient’s insurance status when providing care, or if they worked at both a faculty practice and a hospital-based clinic, did not need to “run back and forth every day” between multiple practice locations. Participants also frequently expressed that it feels better to provide care in a unified practice compared to a segregated one: “I never wanted to look at the patient’s face sheet and know what insurers they have… I just want to take care of the patients.” One participant described this factor as the “moral compass,” arguing that segregated care “wasn’t consistent with our [the institution’s] values” and that a unified practice feels like a more equitable approach to medical care.

### Challenges of Unification

Participants also cited financial concerns as a potential challenge after unification. These concerns were related to reductions in the proportion of revenue received by the department following unification, even as overall collections increased. Additionally, some participants noted that after unification, patients receive 2 bills after a care visit: a professional bill and a facility bill. While most participants considered this a minor challenge—“I have to say that all those complaints disappear…probably it’s because people understand”—, others worried that if patients have the option to receive care elsewhere, they “would leave in a heartbeat” to receive just a single bill.

Interviewees also stated that some staff members struggled to adjust to new roles when a unified practice integrated nonunionized with unionized employees who often have a stricter scope of work and were not permitted to perform previous activities such as phlebotomy. Additionally, participants working in completely unified practices described challenges interfacing with practices that were not yet fully unified. For example, considerations that were irrelevant in their unified practice became salient when referring patients to more segregated specialties. At times, this led to more disjointed care because practitioners experienced less control over when, where, and from whom patients receive specialty care, and consequently, were “not providing the same level of care… that [they] would otherwise, when [they] know the [practitioner] who is going to see the patient.”

Lastly, some participants described negative patient reactions after unification, noting that some patients who historically frequented care sites with only White and/or commercially insured patients were uncomfortable sharing a waiting room with a diverse patient population, expressing that they did not want to “sit with those people.” In response to this challenge, a participant described hiring ambassadors whose job it was to “make patients [with varying needs] feel comfortable” in the shared waiting room.

### Incomplete Unification or Partial Segregation

Although all participating practices were identified as unified practices because they accepted both Medicaid and commercially insured patients, some practices remained partially segregated. These clinics accepted both commercially and Medicaid-insured patients but had different practitioners or schedules for patients covered by Medicaid compared to those commercially insured. Movement toward a fully unified status was inhibited by lack of available space and attending physicians’ capacity for providing care. At these sites, interviewees stated that unification efforts at these clinics improved education, staffing, and/or care quality but as one participant claimed, “until that space is provided, it [complete unification] won’t happen.”

Based on the factors identified, we generated a flow diagram depicting the path from a completely segregated clinic to a completely unified one (Figure). It also includes some interim steps that manifest as partial segregation.

**Figure.**
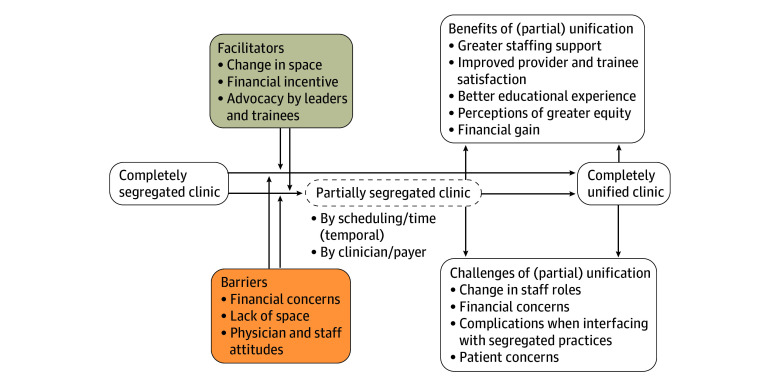
Barriers, Facilitators, and Effects Associated With the Path to Clinical Practice Unification

## Discussion

We explored clinical, administrative, and health system leaders’ perspectives on the barriers and facilitators that prevent or enable practice unification, and the positive and negative effects associated with unification. Our findings suggest that complete practice unification requires consideration of 3 critical factors with the potential to both encourage and discourage unification in different situations: financial, spatial, and attitudinal. Furthermore, we found that not all unification efforts were entirely successful. In some practices, there were persistent dimensions of segregation—by practitioner, payer, scheduling, or timing. This complexity, in which segregation manifests in multiple and layered contexts based on practitioner, payer, and geography, is consistent with reports from existing literature^6,18^ and will require multipronged approaches to achieve equity.^19^

Financially, there are pros and cons to unification because reimbursement differentials among insurance plans are fundamental to the challenge of unifying practices. On the negative side, Medicaid reimburses at lower rates than other insurers^18^ and imposes basic structural challenges to clinic unification.^20,21^ Therefore, maintaining a practice’s financial viability requires a mix of insurance types. Physician reimbursement in faculty practices is often driven by the volume and payment schedules of the commercially insured patients. Thus, moving commercially insured patients to another clinic may negatively affect individual physician salaries, making physician recruitment and retention more challenging.

On the positive side, government programs such as the 340B Drug-Pricing and New York State’s Article 28 promote financial feasibility.^22^ Although participants frequently cited the role of 340B payments in enabling unification, challenges remain. Drug pricing via the 340B program provides benefits to specialties that use more of the eligible drugs than to other specialties or primary care practices. Unification may not be financially feasible for specialties without 340B payments. It remains to be seen whether hospital leadership can create an equitable approach that uses 340B funds to facilitate integration of all clinics. Of concern, ongoing legal disputes have introduced uncertainty regarding the long-term feasibility of the 340B program.^23^

Each US states’ payment reforms can also drive equity-focused changes such as practice unification.^11^ New York’s Article 28 has the potential to facilitate or thwart unification and improve reimbursement but only in specific physical settings that may be unavailable or unaffordable. For example, Article 28’s stringent building requirements likely unintentionally deter interested applicants wary of the added construction expenses. Several states have designed innovative approaches to integrate equity with reimbursement programs, which may act as valuable inspiration.^24^ The Centers for Medicare & Medicaid Services offers states opportunities to develop multipayer models to address health equity.^25^ The challenge, particularly in times of inflation and low tolerance for tax increases, is financing these initiatives. In addition, it is important to monitor the impact of federal and state programs intended to enhance equity but that may inadvertently create barriers.

Regarding space, the feasibility, availability, and cost of providing spaces that can accommodate the larger requirements of Article 28 and a larger volume of combined commercially and Medicaid insured patients emerged as a major factor in this study. Our findings are heavily impacted by our health system’s densely populated location, a geographic factor that encompasses state-specific benefits, such as the financial gains afforded by Article 28 policies, and also imposes spatial requirements that imply costly construction. In states with total cost of care (TCOC) models that are not fee-for-service or volume-based, clinic unification can depend on aligning financial incentives, effective technology use, shared patient care models, and strong leadership. Even within a TCOC model, important challenges, such as data fragmentation, cultural resistance, and regulatory obstacles, remain. Further research should investigate whether space remains a primary facilitator and/or barrier to unification in less populated areas, and how other state-specific or national reimbursement policies could better promote the geographic unification of practices.

Lastly, in terms of attitudes and advocacy, leadership, practitioner, administrative, and patient attitudes can serve as motivators and hindrances to clinic unification.^6,9,10,26^ Several interviewees underscored the insistence of students, trainees, and physicians to unify clinics because “it’s just the right thing to do.” Yet, there were also echoes of classism,^6^ as others expressed concern about patients who would react poorly to sharing a waiting area with people who are “very unkempt.” History teaches us about the value of strong leadership in pushing unification efforts forward, as seen in military^27^ and school desegregation efforts.^28^ In fact, this study found that the first practices to unify were those mobilized by hospital or department leaders.

Among practices that had accomplished some degree of unification, participants felt that unification was largely a positive change. Participants also reported benefits related to staffing support, practitioner satisfaction, trainee educational experiences, and when reimbursement policies were favorable, overall profit. Remaining unification challenges related to logistical nuisances associated with any change in practice space, finances, norms, and structures. As more practices unify, and unified practices refine their operations, the impact of changing staff roles, the issue of interfacing with segregated clinics, and patient concerns are likely to decrease. Indeed, a participant noted that patients who were initially frustrated by changes imposed by unification adjusted to new policies within a few months.

### Limitations

This work has some limitations worth noting. The study was conducted within a single health system in New York City, a city with relatively generous Medicaid reimbursement and state-specific strategies, which may not apply to other locales. However, policymakers often look to other states for innovative health care equity and payment strategies.^25^ Also, the number of interviewees is small, but we interviewed leadership of all unified clinics in a large urban health system. Patient perspectives on unification were not sought during this study phase but are planned for future phases.

## Conclusions

This qualitative study found that clinic unification is possible when efforts are supported by hospital leadership and national and state reimbursement policies. However, these policies are vulnerable to political and regulatory challenges, putting future unification efforts at ongoing risk. Pending concerted efforts to fortify equity in Medicaid reimbursement, clinical and administrative leaders can work to harness existing reimbursement opportunities to support clinic unification and bravely lead us to more equitable and unified practices. In the interim, it is important to be aware that not all practices that are seemingly unified have eliminated structural differences in care delivery. Delineating the impact of structural differences on care quality will be critical to sustaining progress toward truly unified practices.

## Notes

Supplement. Data Sharing Statement
