# Omitted Additional Contribution

**DOI:** 10.1001/jamahealthforum.2025.0690

**Published:** 2025-04-04

In the Original Investigation titled “Unifying Outpatient Practices to Redress Structural Racism in an Urban Health System”^1^ published online on February 21, 2025, in *JAMA Health Forum*, the Additional Contributions section omitted 3 members of the External Advisory Board. The article was updated online.
