# Supplementary material for: Unifying Outpatient Practices to Redress Structural Racism in an Urban Health System
Source: JAMA Health Forum. 2025 Feb 21;6(2):e245520. doi: 10.1001/jamahealthforum.2024.5520 (PMC11846003; doi:10.1001/jamahealthforum.2024.5520)
Supplement: Supplement. — Data Sharing Statement [file jamahealthforum-e245520-s001.pdf]

Data Sharing Statement

Schlozman. Unifying Outpatient Practices to Redress Structural Racism in an Urban Health System. *JAMA Health Forum*. Published February 21, 2025.
doi:10.1001/jamahealthforum.2024.5520

Data
Data available: No
